# Postdigital Learning for a Changing Higher Education

**DOI:** 10.1007/s42438-022-00307-2

**Published:** 2022-05-02

**Authors:** Joan Ball, Maggi Savin-Baden

**Affiliations:** 1 Tobin College of Business, St. John’s University, NY Queens, USA; 2 School of Education, University of Worcester, Worcester, UK

**Keywords:** Postdigital learning, Liminal space, Change, Transition, Disjunction, Higher education, Ecotone

## Abstract

This article draws upon the authors’ previous work on the opportunities and challenges learners face as they move into, through, and out of transitional learning spaces. The authors argue that equipping students to locate themselves with/in the tensions of learning is imperative, especially in postdigital learning environments. Teaching in ways that enable students to acknowledge their ‘will to learn’ and to understand that liminal learning can prompt distress and disjunction or the ability to think differently is discussed. This article begins by examining the relationship between the postdigital and the liminal, offers an Integrated Model of Transitional Learning Spaces, and presents different types of disjunctions. It then explores how learners navigate the liminal and concludes by arguing for a need to focus on postdigital learning and liminal learning in a changing higher education landscape.

## Introduction

The postdigital is defined here as a stance towards the digital which seeks to challenge the educational, economic, and ethical impact of digital technology on humanity and the environment. For example, while learning at universities through digital technology in the past has been seen as largely supplemental, it now takes center stage. The original drive to position the postdigital was in the late 1990s. Such positioning stemmed from the necessity of considering the impact of the (new/er) technologies on existing conceptions of posthumanism, artificial intelligence, and the digital. It could be suggested that the drive for the postdigital began following the argument by Negroponte (1998) that the digital revolution was over. However, as Taffel (2016) argues after the period that was seen to be a digital revolution according to Negroponte, Internet traffic increased as did global users, suggesting that any kind of global revolutions stopped after 2008 is unlikely. However, early uses of the term postdigital were also used to stand against a binary stance towards the digital, suggesting instead it should be seen not as an either other but as a continuum. For example, Pepperell and Punt suggest:

The term Postdigital is intended to acknowledge the current state of technology whilst rejecting the conceptual shift implied in the ‘digital revolution’ – a shift apparently as abrupt as the ‘on/off’ ‘zero/one’ logic of the machines now pervading our daily lives. (Pepperell and Punt 2000: 2)

While some authors in the 2020s would argue that we are no longer in the postdigital (for example, Cramer 2015), we argue that the constellation of stances that comprise the postdigital condition represents a liminal and disruptive space in which to untangle the impact of the digital on diverse systems and relationships. This critical perspective, a philosophy, can be summarized as:

- A disenchantment with current information systems;
- An exploration of digital cultures that is both still digital and beyond digital;
- The blurred and messy relationship between humanism and posthumanism;
- The condition of the world after computerization;
- The state of global networking and its development;
- The expansion of the digital market.

Thus, we argue that the postdigital is neither temporal nor ‘after’ digital; rather, it is a critical inquiry into the state of the digital world that is characterized by its ungraspability. This ungraspability relates to the way in which structures, political systems, cultures, languages, and technologies intersect to change each other and the state of the world. As such, the postdigital describes a state of becoming where the human and the digital are interacting, co-creating, and merging in ways that are beyond imagining. According to Savin-Baden (2021), postdigital humans are located in this liquid, non-linear space, and play a key role in the formulation, interruption, and (re)creation of information and learning systems. Existing within these spaces as they morph, change, and evolve contributes to the perceived ungraspability of the postdigital, and suggests that postdigital learning is marked by uncertainty, liminality, and mystery that can feel threatening, at worst, and transformative, at best. Thus, developing opportunities and experiences to equip learners to navigate the liminal and engage with liquid learning spaces is fundamental to flourishing in the postdigital.

Although we seek to define postdigital learning and forms of liminality, we are also concerned about over defining student identity and essentializing learner experience. Although this is explored in more depth in the discussion, we wish to stress at the outset that the exploration of disjunction, liminality, will-finding, and wayfinding are meant to be seen as concepts to prompt thought. What we mean by this is that such concepts can enable educators to understand the complexity of spaces students inhabit, how students develop a will to learn, how they come to understand themselves, and the ways in which their student identities are bound up with personal and social values, and how this influences their responses to disjunction and liminality.

## Liminality and Liquid Learning Spaces

Liminality is a term first coined by van Gennep who described a psychological or metaphysical subjective state of being at the threshold of two existential planes. Although the term was originally applied to rites and rituals in small human groups, it was extended to whole societies by writers such as Jaspers (1953). Turner later described people in a liminal state as ‘a realm of pure possibility whence novel configurations of ideas and relations may arise’ (1995: 97). He suggested that those in liminal states were often ritually, symbolically, or metaphorically removed in order not to threaten the social order while they experience transition, transformation, or ‘in-betweenness’.

In the transition from what was traditionally a largely analog delivery of information and knowledge from human to human without digital intermediation, educators, learners, and technology find themselves interacting in a new, uncharted territory. Learning in the liminality of the postdigital can disrupt identities and sense of self-direction, which can lead to feelings of insecurity, threat, and epistemological, conceptual, and ontological ‘lostness’. In the context of academic life, for instance, many academics verbalize stories about liminal identities in the context of the personal costs of role transition into and through academe as well as undertaking a PhD. This transition through liminality brings with it not only new knowledge and understanding for the participating individual, but also often new status, identity, and orientation within the community. Yet, to date, there is relatively little understanding of what occurs in the liminal tunnel that exists between the catalyzing disjunction and the new sense of self and self-direction that emerges on the other side.

The liminal tunnel, as described by Land et al. (2014), begins with a ‘portal or gateway triggered by the threshold concept or disjunction’. Learners move through the tunnel and emerge with a shift in learner subjectivity, a discursive shift, or a shift of a conceptual, ontological (such as identity shifts), or epistemological nature. Land et al. (2014) depict this transformation as a cognitive tunnel where the liminal space within the tunnel is entered when triggered by a threshold concept, or a ‘disjunction’, that challenges previously held ideas about something. Disjunctions are ‘spaces’ or ‘positions’ which are reached through the realization that knowledge is troublesome. For instance, after encountering a threshold concept, the learner will move into a liminal space that can be transitional and transformational. Learning in the liminal space often entails oscillation between different states and emotions. The liminal space is characterized by a stripping away of old identities, oscillation between states, and personal transformation (Savin-Baden 2008). Yet these liminal zones are not to be seen as dead, wasted, or terrible stuck places but instead places of growth. There is often a sense that liminal spaces or tunnels, in which these liminal zones exist, are abandoned lots or graveyards.

**Fig. 1:**
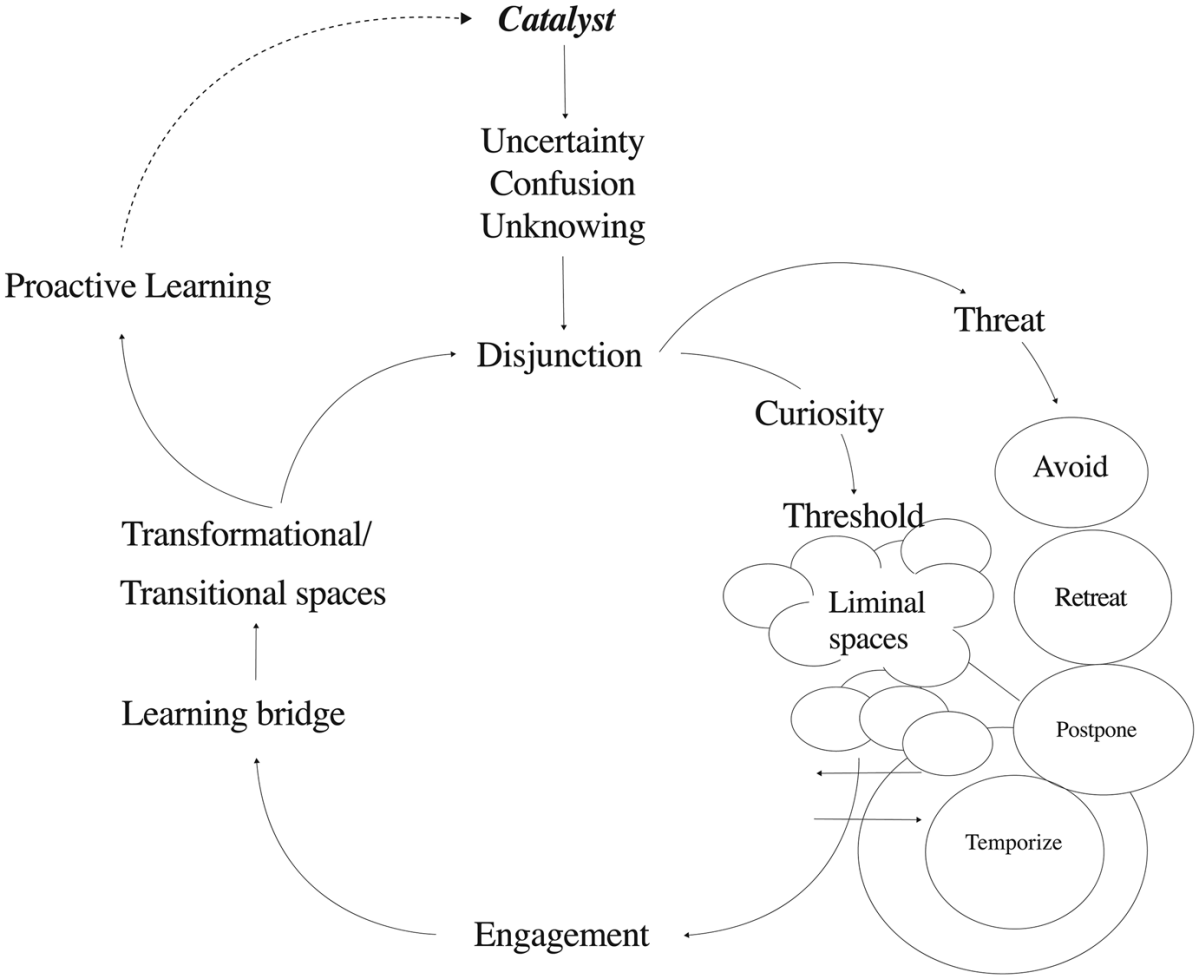
Integrated Model of Transitional Learning Spaces

The postdigital is seen here as liminal learning space, a tunnel in which to untangle the impact of the digital on economic, sociological, political, and ethical systems and relationships. Yet it is not clear how or even whether this liminality contributes to the ungraspability of the postdigital, or indeed if understanding more about learner and educator experiences in the liminal tunnel provides some context for living and learning in the liminality of the postdigital. What is known is that some catalysts to change can prompt feelings of uncertainty, confusion, or a sense of unknowing that results in disjunction and/or a feeling of stuckness. The difficulty with disjunction is that explanations of its forms, and possibilities for moving away from them, do not always enable people who are stuck to enter liminal space, and instead they stand stuck on a threshold. The result is that learners can see disjunction as a threat. A response to threat at the point of disjunction might be to seek to regain control and place boundaries around disjunction through avoidance, retreat, postponement, and temporizing (Savin-Baden 2008). If the disjunction generates curiosity, on the other hand, or a desire to learn (by choice or necessity), learners cross the threshold into the liminal tunnel where they explore new information, knowledge, and experiences (Ball 2022). Once through a liminal space the journey continues through engagement, over a learning bridge and on to a position of transition or transformation, and then to a place of proactive learning, as illustrated in Fig. 1.

Many staff and students have described disjunction as being a like hitting a brick wall in learning. Disjunction is similar to troublesome knowledge because until it is experienced in a learning environment it is difficult to explain, particularly in terms of students feeling fragmented. Perkins’ work is useful here in understanding the impact of diverse forms of knowledge, and how this is often covert and therefore misunderstood by students, and also often disregarded by staff. Perkins (1999) described conceptually difficult knowledge as ‘troublesome knowledge’. This is knowledge that appears, for example, counter intuitive, alien (emanating from another culture or discourse), or incoherent (discrete aspects are unproblematic but there is no organizing principle).

Disjunction is not only a form of troublesome knowledge but also a ‘space’ or ‘position’ reached through the realization that the knowledge is troublesome. This experience of disjunction as both a ‘state of being’ and a ‘space of being’ is not unlike how humans experience the postdigital—as both a state of potentially troublesome educational, economic, and ethical impact of digital technology on humanity, systems, and the environment; and as a potentially troublesome liminal space where humans and the digital are interacting and co-creating in the (re)creation of information and learning systems. Thus, in the same way that disjunction might be seen as a ‘troublesome learning space’ that emerges when forms of active learning (such as problem-based learning) are used that prompt students to engage with procedural and personal knowledge, postdigital learning may also be seen as a ‘troublesome learning space’. However, the procedural and personal knowledge necessary to navigate this space is emerging and shifting at the unprecedented speed of evolving technologies.

Therefore, just as disjunction can be seen as a place that students might reach after they have encountered a threshold concept that they have not managed to breach, postdigital learners are bombarded with catalysts to disjunction as they are forced to rethink and reimagine both new and established learning as emerging technology influences both their digital and analog environments, in- and outside of formal learning environments. Thus, having a more nuanced understanding of different types of disjunction or levels of disjunction and how they might result in movement into different kinds of liminal spaces might be helpful for educators tasked with preparing postdigital learners with the skills and approaches they need to be equipped for pervasive and persistent encounters with disjunction. The following forms of disjunction are offered as a stepping off point for considering this more nuanced view.

## Forms of Disjunction

Engaging with disjunction requires learners to acknowledge its existence and attempt to deconstruct its causes by examining the relationship with both their internal worlds (threats to identity and perceptions of self) and external worlds (identity in space/social environments). Ball (2022) argues that understanding and aligning ones internal and external worlds in the face of disjunction present a variety of challenges to emotional, material, and social resources. This presents a complex set of practical threats to learners that has not received adequate attention in the literature. It also points to a gap in our understanding of the variety of forms of disjunction and the resources people need to develop their capacity to navigate into, through, and out of liminal space, especially since the shift into postdigital learning. For example, millennial generation students facing cultural shift and tech disjunction were described as ‘failing to launch’ when they did not engage with emerging adulthood as previous generations had (Arnett 2000). While not a panacea for a complex and multi-dimensional challenge, postdigital disjunction has been observed to be a contributing factor in the stuckness many emerging adults describe at points of inflection. Helping learners to accept the existence and importance of disjunction and enabling them to develop practices to navigate liminal space and engage in liminal learning may help them to endure the ungraspability of the liminal in the postdigital.

While student encounters with disjunction will vary, some varieties might include conceptual, ontological, and epistemological forms of disjunction, as illustrated in Table 1.

**Table 1:** Forms of disjunction

Form of disjunction	Description	Example	Related work
Narrative disjunction	An interruption is someone’s learning journey, resulting in disorientation and a sense of losing one’s way	Student is confident that they have command over an idea or concept and learns mid-way through a presentation that they missed a key element	Ball (2022)
Ontological disjunction	A sense of stuckness related to a need to reinterpret what was once familiar since they now seem strange	In moving from analyzing qualitative data to interpreting it, students can just re-analyze the data and create further themes and lists rather than interpreting it at a level of subtext	Lather (2006)
Conceptual disjunction	When grappling with a new idea the understanding that comprehension is just under the surface of our thinking	Student struggles to understand around an entirely new and challenging concept (e.g., the blockchain)	Entwistle (2008)
Epistemological disjunction	A failure to understand that there are different forms of knowledge and the these have an impact on what is learned and how things can be learned to best effect	Student struggles to engage established learning through new and different framing or lens	Savin-Baden (2008)

### Narrative Disjunction

This is an interruption in someone’s learning journey, resulting in disorientation and a sense of losing one’s way. Often this occurs when a moment of misconception is drawn attention to by someone else, leaving the person concerned both stuck, exposed, and in doubt. For example, at a conference it may become apparent to someone that a concept they believed they had grasped and understood they in fact do not. Narrative disjunction is experienced both privately and in the view of the person or people who point out the moment of misconception. If the external prompt to disjunction leads to a shift in identity and/or causes them to question their capability to learn, perception of threat can be compounded by insecurity or embarrassment. If the external prompt to disjunction is perceived as an invitation to transitional learning, the person can address doubt and uncertainty by engaging the support or expertise of the person who identified the misconception or others to become ‘unstuck’.

### Ontological Disjunction

This form of stuckness results in a reconsideration of the issues that have promoted becoming stuck. For example, after reconsidering the difficulties the position of stuckness is seen differently, and the familiar is seen as new or strange (e.g., Lather 2006). Thus, here there is a sense of reinterpreting what was once familiar and what once seemed whole into a collection of components, some of which are then rendered strange. What may occur in this form of disjunction is a cycle of stuckness where a student needs to move away from a particular position of stuck space, but not knowing how or where to move to results in disorientation and a constant cycle of stuckness which leads to a return to the same stuck space repeatedly. For example, in moving from analyzing qualitative data to interpreting it, students can just re-analyze the data and create further themes and lists rather than interpreting it at a level of subtext. The cycle of stuckness may lead to a shift in learner identity; however, the positional nature of the stuck space may lead to disorientation, a shift in self-direction, or some combination of both. This can lead to a perception of threat and feelings of frustration that perpetuate and exacerbate the cycle. Identifying the root of the cycle of stuckness can help learners to consider context relevant ways to break the cycle and become unstuck. To move away from cycles of stuckness, new routes forward are called for rather than attempts to return to previous ways of operating.

### Conceptual Disjunction

Conceptual disjunction builds on theories from the cognitive tradition which relates to disjunction that tends to be discipline specific concepts, within specific subject learning. This form of disjunction is often characterized by a moment of conceptual puzzlement where the student realizes that they are stuck and is unable to understand how to move forward. Conceptual puzzlement is experienced privately unless the learner chooses to share their disjunction with others. Threat to identity and questioning of the capacity to learn can lead to doubt, diminishing confidence that can perpetuate stuckness. Developing understanding and use of these disciplinary-related concepts is, it is argued, crucial for student learning and knowledge construction, such conceptual disjunction which relates to the literature on threshold concepts. For example, Entwistle (2008) argues that engaging with threshold concepts is related to *conceptual* change and relates his argument to Perry’s conceptions of knowledge (Perry 1970) and Säljö’s conception of learning (Säljö 1979). Thus, it might not be possible to ‘become’ an engineer, lawyer, or economist unless the student has passed over a number of given knowledge thresholds.

### Epistemological Disjunction

This form of disjunction is characterized by a failure to understand that there are different forms of knowledge and the these have an impact on what is learned and how things can be learned to best effect. Knowledge has been defined in a whole host of ways. Gibbons et al. (1994) have argued for Mode 1 and Mode 2 knowledge. Mode 1 knowledge is propositional knowledge that is produced within academe separate from its use and the academy is considered the traditional environment for the generation of Mode 1 knowledge, whereas Mode 2 knowledge is knowledge that transcends disciplines and is produced in, and validated through, the world of work. Knowing in this mode demands the integration of skills and abilities in order to act in a particular context. While this division has been popular and useful to many, it does to some extent reflect some of the problems of Ryle’s (1947) notion of ‘knowing that’ and ‘knowing how’, which tends to both polarize and separate skills from knowledge. Although Mode1 and Mode 2 knowledge are more complex and their position is better argued than that of Ryle, the problem with both of these stances is in the boundary spaces between the two forms of knowledge. Barnett (2004), however, argues for Mode 3 knowledge, whereby one recognizes that knowing is the position of realizing and producing epistemological gaps. Such knowing produces uncertainty because, ‘[n]o matter how creative and imaginative our knowledge designs it always eludes our epistemological attempts to capture it’ (Barnett, 2004: 252).

What is missing from the arguments and formations of knowledge and knowing is not only the way in which the spaces between these forms of knowledge are managed, but also what it is that enables students and staff to make the connections between all of them. It might be suggested that the missing links here are disregarded forms of knowledge. For example, Cockburn (1998) suggests that knowing when to keep your mouth shut and the virtues of tact are forms of knowing that are required in many professions but are not forms of knowing that are made explicit in the academy. Disregarded forms of knowledge then might be termed Mode 4 knowledge since it transcends and overlays Mode 1, Mode 2, and Mode 3 knowledge, forming a bridge across the space between them. However, Mode 4 knowledge is also a mode in its own right, since it involves not only realizing and producing epistemological gaps but also realizing the ways in which these gaps, like knowledge and knowing, also have hierarchical uncertainty. In contrast, Mode 5 knowledge is a position whereby one holds a number of modes together in a complex and dynamic way. Gaps, like knowledge, have hierarchical positions and this makes both the gaps and the knowledge, and the knowing and the knower eminently uncertain and liquid. The modes are set out in Table 2.

**Table 2:** Modes of knowledge

Mode 1	Propositional knowledge that is produced within academe separate from its use and the academy is considered the traditional environment for the generation of this form of knowledge
Mode 2	Knowledge that transcends disciplines and is produced in, and validated through, the world of work
Mode 3	Knowing in and with uncertainty, a sense of recognizing epistemological gaps that increase uncertainty
Mode 4	Disregarded knowledge, spaces in which uncertainty and gaps are recognized along with the realization of the relative importance of gaps between different knowledge and different knowledge hierarchies
Mode 5	Holding diverse knowledge with uncertainties

## Preparing Learners to Enter Liminal Space: Resilience and Hope

The impulse to avoid crossing the threshold into liminality points to a deficit of resilience at the point of disjunction. Ungar (2019) defines resilience in the face of adversity as the ability for people to navigate their way to the emotional, material, and social resources they need in ways that are meaningful to them and help to sustain their well-being. When stuckness and lostness at the point of disjunction are perceived as a threat to identity and self-direction, learners can find it difficult to connect with those resources, resulting in further disjunction. Preparing for this common response to disjunction is a learning capability that is often overlooked until the point of disjunction is reached, resulting in the aforementioned avoidance rather than engaging in a process of finding their way into and through the liminal space.

A wilderness metaphor is helpful here. Consider two people dropped in the middle of a dense wood in a place neither has been before. The first has orienteering training and hikes with their family several times a month. The other lives in the city and rarely, if ever, spends time in the woods. Both are lost. According to Huth (2013), people react to being lost in remarkably similar ways. Whether trained or untrained, people who are lost ‘can all experience as sense of panic brought on by the ensuing disorientation’ (30). In this example, both lost persons might experience a threat response at the point of disjunction; however, the person with orienteering training and familiarity with being in the woods will have a better chance of getting their bearings reorienting themselves in the new terrain than the one who finds themselves both lost and in unfamiliar terrain. Both will face challenges along the journey to clarity, but the former will be better equipped to make their way. However, it is not only having the learning capability to manage the unknown environment, but also recognizing the relationship between the current learning context in relation to previous learning experiences that influences the learner’s perception of whether unfamiliar terrain is off-putting or an invitation to learning.

This is not surprising, since learners’ perceptions of the learning environment and what is expected from them (more than any objective reality) affect what and how they learn (Marton and Säljö 1984; Prosser and Trigwell 1999), for example, whether they mainly seek to absorb and reproduce knowledge or behaviors from facilitators or learning materials; focus on understanding and generating new knowledge; hope to transform their professional lives; or seek to please important gatekeepers. Overloading learners is known to encourage a reproducing (surface) approach to learning, faulty learning, disengagement, or a strategic approach to studying (Entwistle 2009). Learners’ perceptions are also shaped by explicit and implicit messages. Explicit messages include the following:

- The course description (as published and as spoken by tutors);
- A course handbook containing aims or intended learning outcomes;
- The learning materials and assessment requirements.

These all convey the ‘target understanding’ (Entwistle and Smith 2002) that curriculum developers, tutors, and examiners have in mind (and any lack of alignment can create confusion). Implicit messages, the ‘hidden curriculum’, include the following:

- Perceptions of the importance of a particular learning opportunity based on, for example, who chooses to attend or otherwise contribute and the attitudes they display;
- Any attendance or assessment requirements;
- Whether the event is allocated a bright and airy room with access to adequate technology and refreshments;
- Whether the event is pushed to the fringes of the timetable such as late on Friday afternoon.

When learners stand at the threshold of postdigital learning spaces, the lack of clear expectations or destination may contribute to them perceiving disjunction as a threat. This is compounded when students are intentionally placed in circumstances where the learning environment is not stable, such as groupwork where the location and structure for engagement is uncertain and fluid, where boundaries are blurred (Wardak 2022), or in online learning space with which they are not familiar. Such ‘nomadic work’ experiences (see Rossitto and Eklundh 2007) have the potential to compound disjunction due to a lack of stable and fixed location to carry out their work together. This was evident during the rapid shift from traditional classrooms to online engagement for students across the globe during Covid-19 lockdowns. The blurred traditional boundaries between home and learning space required students to orient themselves in learning spaces that were neither home nor University—a third space—in which they were required to establish new ways of engaging with their work, their instructor, and one another. Equipping students to identify the means and methods to get their bearings, to reorient and resource themselves for the journey across these sorts of liminal space, and to engage with new knowledge is imperative to postdigital teaching and learning. Thus, like the well-trained hiker, learners can then learn to view disjunction and liminality as part of the learning process across contexts, and educators could better prepare them to resource for resilience prior to disjunction. This ‘active resilience’ (Ball 2022) prompts learners to examine their relationship with their internal and external worlds prior to disjunction, increasing the likelihood that learners might learn to view the threshold from disjunction to liminality as an invitation to curiosity, learning, and exploration rather than a threat. This invitation, however, can be another catalyst to disjunction if the learner is not properly prepared and/or lacks the necessary resources for the exploratory journey into liminal space.

For the learner, gaining one’s bearings and reorienting in the face of being lost require hope that a way forward is possible. The relationship between hope and performance has been established across a variety of domains, including academic achievement (Snyder et al. 2002), physical and mental health (Rasmussen et al. 2017), survival and coping (Stanton et al. 2002), and well-being (Chang and DeSimone 2001). While it has been viewed in the literature as a trait (Snyder et al. 2002), more recently hope has been viewed as a developmental state that involves having at least one future goal, the belief that one has the agency and resources to achieve the goal and at least one caring person to support their efforts (Lopez 2013).

Snyder et al. (2002) describe hope as having three components: (1) having goal-oriented thoughts; (2) developing strategies to achieve goals; and (3) being motivated to expend effort to achieve goals. Luthans et al. (2015) describe hope as a positive motivational state where two basic elements—successful feeling of agency (or goal-oriented determination) and pathways (or proactively planning to achieve those goals)—interact. They refer to these concepts as will-finding and wayfinding, defined here as follows:

- Will-finding is the realization of the importance of the will as a central plank of the learning journey;
- Wayfinding is a realization and sense of hope that there is an accessible path through the liminal tunnel and the learner can connect with meaningful resources to find it.

Barnett (2007) argues for the difference between motivation and the will to learn as being an important concern in higher education. Motivation is deliberate—the desire to run away from a tiger to prevent from being eaten, he suggests. Thus, while student motivation to learn may form a desire to move away from home or gain a good job, the will to learn is internal and ontological and, we would suggest, it is central to identity. Will, then, is an important issue in the management of disjunction, as it precedes the process of wayfinding or requirement and inspires an existential moment when learners enter liminal learning space by the force of their own daring. The will is vital to movement through liminality in order to move or even leap through the tunnel, as Barnett suggests:

There is also the existential moment in which the student leaps into those experiences with her own daring. It is a bungee-jumping moment . . . This leap of becoming is – again, just like the bungee jumper – paradoxical: it is a leap into a void, into a new space, but it is, at the same time, a leap by the student into herself . . . Because she has willed herself into that space, she can identify with her new self in that space. There is personal satisfaction to be obtained here. This is not just a new becoming; it is becoming itself . . . The student did not know how it would be before the leap and now, having come into the new place, still cannot be sure of its validity. However, she has won this space and this place for herself. Through the learning processes, which she has undergone, she is able to defend her new place. (Barnett 2007: 54–55)

Moreover, having entered and exited the liminal learning space, the learner carries with them the experience of having successfully navigated uncharted territory, and can bring that experience and confidence to the next liminal learning experience. Thus, when faced with disjunction in the future, the process of goal setting and belief that one has the agency and resources to enter and traverse liminal space can be developed and practiced. It can also be compromised, especially when the learner does not have social support to help them to navigate transitional learning space. Thus, the learner can lose their will and their way through liminal learning space, perpetuating further disjunction. As with the development of ‘active resilience’, this view of will-finding and wayfinding as a means of engaging a hopeful view of disjunction suggests that developing the will to learn at points of disruption and interruption can be engaged as a means of building capacity to deal with postdigital learning. Part of the will to learn is hope.

Contextualizing hope as will-finding and wayfinding provides educators with a framework to help learners to access the resources they need to enter and navigate liminal space prior to their experience of disjunction. It also suggests that learning to engage with disjunction and liminal space may require learners to identify interim goals designed to enable them to reorient themselves, gain a sense of agency, identify new pathways, and connect with a social support network which may not be directly related to their initial learning intention. Thus, navigating the liminal may exist beyond particular learning intentions and be accessed as a result of previous learning contexts and experiences which may prompt an enabling response to disjunction, or a liminal identity, that can be called upon to light the way into, through, and beyond the liminal tunnel when needed.

## The Rhizomatic Tunnel and Liquid Pathways

Field (2012) argues for the idea of ‘liminal identity’, the notion that such an identity can be shaped through cultural and social processes that are formed and challenged through relationships with others. However, in terms of postdigital learning, it is not clear whether they are imposed from the outside or something over which people have control and choice. In a recent study (Fredholm et al. 2020), data were analyzed using the theoretical representation of the cognitive tunnel (Land et al. 2014). Students’ narratives described their disjunction, their experience of the liminal spaces, and their resulting shift over the thresholds. Instead of focusing on a cognitive tunnel as Land et al. (2014) suggest, this was related to a particular practical experience functioning as a trigger for moving into the tunnel, learning in the tunnel, and coming out ‘on the other side’ of the tunnel with a changed view. The driving forces for movement through the tunnel were the students’ inner motivations for learning, originating from the perceived meaning of the practical experience. The self-evident nature of the practical experience and the need to master these situations created movement and transformational learning. Table 3 depicts movement into, through, and out of the tunnel with triggers and consequences.

**Table 3:** Depiction of movement into, through, and out of the tunnel (adapted from Fredholm et al. 2020)

	**Triggers to movement**	**Consequences**	**Interventions**
Experiencing disjunction	Disjunction in the form of a conceptual, epistemological, or ontological experience	Confusion, stuckness, and frustration; Challenge to previously held beliefs	Pause to acknowledge and temper emotional reaction to disjunction
Hovering on the threshold of the liminal tunnel	Fear and/or curiosity about unknown opportunities and challenges to learning in the uncertainty of the liminal tunnel	Avoidance, retreat, postponement, temporizing, or engagement	Gather resources and supports to manage emotional response to disjunction and intentionally shift from perception of threat to curiosity about the liminal tunnel
Entering the liminal tunnel	Movement triggered by hope and curiosity	Inspiration to learn and motivation to explore	Focus on will-finding and wayfinding
Learning and developing in the liminal tunnel	Surprise, adaptation, and recognition of need to learn and shift position and then reorient identities as learning uncovers new information and shifting terrain in the liminal tunnel	Transitional and sometimes transformational learning; Gaining self-direction	Explore new possibilities and seek to experiment as means of understanding the contours of the liminal tunnel and potential routes through and out of it
Moving toward the end of the tunnel	Sudden or gradual understanding, a stripping away of old identity, gaining a sense of direction and personal transformation	A conceptual, epistemological, or ontological shift evident in change in any or all personal, professional, and learner identity	Make sense of options, intentions, and potential outcomes
Hovering on the threshold of the exit	Acknowledgement of potential routes forward and discern among them to identify possible futures beyond the liminal tunnel	Can lead to fear, uncertainty, and perception of threat	A shift from exploration, liminal learning, and identifying options toward choice-making and execution
Crossing the threshold and exiting the tunnel	Confidence gained though threshold shift	Seeing the world afresh and valuing the disjunction and subsequent shift	Choice-making and execution

It is proposed here that the liminal tunnel is not merely cognitive as Land et al. (2014) suggest, but ontological and rhizomatic. Previously, however, Meyer and Land (2006) have explored the idea of the liminality as an ontological and cognitive space, discussing the ways in which identity is reconstituted through language as students encounter threshold concepts. The example used in their study is that of a French student discursively presenting themselves as a French speaker. However, to date there has been relatively little exploration of types of tunnels, or indeed what occurs inside them.

Liminal spaces within the liminal tunnel are suspended states and serve a transitional, and sometimes transformative, function as someone moves through the tunnel. Within the tunnel, people begin to re-examine their position, which is not just cognitive and epistemological but ontological, and in doing so see the terrain that they then choose to move through towards the end of the tunnel. For most people, the concept of a tunnel is invariably imagined as a narrow one-directional space. In the context of liminal ecologies of learning, tunnels are rhizomatic. The rhizome, in Deleuze and Guattari’s terms, is a cultural model based on the botanical rhizome. It is positioned in opposition to a root-tree system which follows chronological lines, and which looks towards pinnacles or conclusions. By contrast, the rhizome is always interconnected, and ‘has no beginning or no end; it is always in the middle, between things’ (1988: 25). Some liminal tunnels can be one-directional; these tend to be either rites of passage or temporary disjunctions which result only in a series of transitions, rather than fundamental transformations. Thus, these tunnels are temporary, in which the eventual end point or the crossing of a threshold concept boundary is the focus. If rhizomatic tunnels are where transformations occur, we suggest that postdigital learning represents a rhizomatic tunnel that holds potential (and necessity) for transitional and transformational shifts in learning, doing, and being.

## Discussion and Reflections on Liminal Learning in the Postdigital

The difference between transitional and transformational shifts through liminal tunnels is that in a transition there is a sense of shifting from one place to another, whereas in a transformational there is a sense of life-shifts, of knowing the world differently in living, working, and learning contexts. For example, spending 4 or 6 years in university can be transitional if a learner completes their term having not explored who they are and how they fit in the world. On the other hand, university can be incredibly transformative when a student engages in a process of deepening their knowledge of who they are, where they are, what is meaningful to them, and how they might bring that meaning to bear on the world in which they live.

This difference between a transactional view of higher education (e.g., trading time for a diploma) and a transformative view of higher education (e.g., a time of exploration) is profound in the context of postdigital learning, since particular degrees and diplomas are becoming less important than the capacity to change, learn, and adapt a changing landscape across the lifetime. It is important, therefore, to take a critical stance towards the shifts into and through the liminal tunnel since the subsequent transitions may or may not result in transformations and transitions into different forms of learning fluency. Daignault’s work is helpful here since he argues for performing ‘knowledge through a passageway’ through thinking aloud (1983: 7–13). The idea is that the gap is the curriculum and of what creates the curriculum is a composition of thinking and wisdom, ‘thinking maybe’ (1992: 202). Daignault’s work is not about crossing thresholds, but walking in between, and we would suggest, walking along the edges of the rhizome. Thus, a curriculum, learning space, or any education experience becomes a creation and a composition, a thinking space that is complex and multi-layered. A third space of sorts. This notion of a third space can be a helpful way to think about the liminal space between the time prior to a catalyzing disruption that leads to disjunction and the crossing of the learning bridge. An example from biological science provides a helpful framework to think about these third spaces.

The term ‘ecotone’ is borrowed from biological science, which defines it as a transitional area between ecosystems where ‘edge effects’ such as new species and unpredictable outcomes can be observed (Graves et al. 2011). Introduced in the early part of the twentieth century as ‘a stress line connecting points of accumulated or abrupt change’ (Slater 2016), the ecotone provides a useful metaphor for the new and sometimes unpredictable, even paradoxical, third space at the intersection of analog, digital, and postdigital learning spaces. This third space between identity before and identity after passing through a transitional learning space is well documented (Kiley and Wisker 2009; Land et al. 2014). The original notion of the third space captures the idea that there are ‘particular discursive spaces ... in which alternative and competing discourses and positioning transform conflict and difference into rich zones of collaboration and learning’ (Gutiérrez et al. 1999: 286–287). These spaces tend to be polycontextual, multivoiced, and multiscripted. Although the research by Gutiérrez et al. relates to children learning across languages and cultures, the notion of third spaces is helpful in locating and understanding the languages, discourses, and cultures implicit in postdigital learning.

This may contribute to the ungraspability of the postdigital and underscores the proposition that equipping students for liminal learning is a postdigital learning imperative. It is important to note that drawing from the biological sciences to describe learning in the postdigital condition as an ecotone is not without its challenges. Ryberg et al. (2021), for instance, acknowledge that, while ecotones as a conceptual metaphor have the potential to expand postdigital thinking, they distinguish between ecotones and liminality as distinct in place, process, and orientation toward transition and transformation. While this nuance has the potential to complicate the use of the ecotone metaphor to describe transitional learning space (which can also, but not always, be transformational), it is our view that the nature of transitional learning spaces may be informed by both concepts. Thus, in the same way that the ecotone that exists where a meadow, a river, and a forest meet might produce new, and sometimes competing, plant and animal species (Graves et al. 2011), the postdigital reflects the sometimes ambiguous and changeable liminal space, wherein multiple priorities, emotions, goals, and other influences converge to create a new learning ecosystem.

Here, then, we consider the notion of a postdigital learning ecotone that is forming, reforming, and evolving with changing technology in ways that are varied, unpredictable, and, at times, counterintuitive. Postdigital learning, then, needs to encourage students to interrogate both new knowledge and the learning spaces in which they are delivered: the striated managed university spaces that need to be interrupted, whether in the UK, the USA, Asia, or the Global South. Furthermore, the internationalization of the curriculum should not be about the learning for commercial gain (Wimpenny et al. 2020) but the development of inclusivity, equality, and critical pedagogy in a variety of higher education learning spaces. Learning a module of some subject is no longer enough. What matters is shaping learning so that it enables students to engage in participatory politics, and networked publics, to undertake problem management and to become digital citizens, whose brinkmanship is based on a desire to mess around in order to understand and transform their learning lives in liquid ways, so that learning leaks across the various boundaries of their worlds. This may involve learning in less structured environments (e.g., liminal spaces and ecotones) and prompts creative and innovative approaches to uncertainty and change.

After someone has first encountered disjunction, they enter a liminal space. However, it might be that there are different types of disjunction or levels of disjunction, and that these might result in movement into different kinds of liminal spaces that require different capacities, skills, and approaches to navigate in order to flourish. In this sense, approaches to postdigital learning which encourage engagement with flexible pedagogies (Barnett 2014) and criticality will result in transformational learning and prompt learning fluency and open routes to human flourishing in the postdigital. Flourishing then is about disjunctive affirmation, as Lather explains in the context of research methods teaching:

I have endorsed a ‘disjunctive affirmation’ of multiple ways of going about educational research in terms of finding our way into a less comfortable social science full of stuck places and difficult philosophical issues of truth, interpretation, and responsibility. Neither reconciliation nor paradigm war, this is about thinking differently, a reappropriation of contradictory available scripts to create alternative practices of research as a site of being and becoming. (Lather 2006: 52)

Perhaps learning and the development of fluency in learning demand the ability to live and learn liminally, moving adeptly from liminal learning spaces to learning bridges and back again. Such thinking spaces are not narrow and linear, but complex, multidirectional, and multi-layered, similar to Corner’s (1999) mapping practices which he names drift, layering, and rhizome. Such curricula will encourage rhizomatic travel since the curriculum itself is a liminal learning space.

## Conclusion

Teachers need to practice such disjunctive affirmation in order to ensure students continue to understand the importance of disjunction as a component of finding both their will and their own creative and exploratory ways to learn in liminal and non-liminal spaces. To get there, educators, researchers, and digital produce developers need to pursue new lines of inquiry that explore more deeply the relationship between liminality and postdigital learning. Moreover, a more nuanced view of the relationship between different forms of disjunction and the particular kinds of disjunction that face postdigital learners is called for. And finally, if will-finding and wayfinding are key capacities for navigating liminal and postdigital learning spaces, more information about what is happening in the rhizomatic tunnel and how to uncover liquid pathways into through and beyond the unknown is critical understanding if we are to equip future generations to flourish in the postdigital.
